# Peer Review of “Cardiotoxicity in Pediatric Cancer Survivorship: Retrospective Cohort Study”

**DOI:** 10.2196/79521

**Published:** 2025-07-31

**Authors:** Archana Adhikari

**Affiliations:** 1 Sikkim Manipal University, Gangtok, India

**Keywords:** cardiotoxicity, cardiology, cardiovascular, heart, arrhythmias, self-reported questionnaires, oncology, survivors, pediatrics, prevalence, incidence, risk, epidemiology, anthracycline exposure, childhood cancer survivors

*This is a peer-review report for “Cardiotoxicity in Pediatric Cancer Survivorship: Retrospective Cohort Study.”*

## Round 1 Review

### General Comments

This paper [1] gives valuable insights into cardiotoxicity in pediatric cancer survivorship: patterns, predictors, and implications for long-term care. The results and methodology are sound. However, some minor revisions would improve clarity and strengthen the overall impact of this paper. Below are my suggestions.

### Major Comments

1. Method section (study population and data source): In the Method section, specifically the fourth line, the description “of 21 at one of 31 participating institutes” is unclear. The sentence should be revised for better clarity.
2. Missing answer for seventh objective: The answer to the seventh objective is unclear.

### Minor Comments

1. Result presentation: It would be better if the results were presented in tabular format for easier comprehension. A table would help summarize the key findings and increase readability.
2. Clarity in results numbering: To improve clarity, it would be beneficial to present all the results with corresponding numbers, matching each result with the respective objective number for easier reference and alignment.
